# Patient epidemiology in a level II hospital ICU and how main nosocomial infections affect morbidity and mortality

**DOI:** 10.1186/cc14174

**Published:** 2015-03-16

**Authors:** M Muñoz, E Yuste, O Moreno, R Fernandez, R Ramirez

**Affiliations:** 1 Hospital Universitario San Cecilio, Granada, Spain

## Introduction

We describe the type of patient and the main nosocomial infections in a level II hospital ICU unit, 18 beds (12 polyvalent-general, six coronary).

## Methods

We used the ENVIN-HELICS database and made statistical calculations for all patients admitted to the ICU between 1 October 2012 and 30 September 2013 using SPSS v.15.

## Results

Patients admitted (1,126): 65.1% were male; mean age 61.72 (SD ±15.8), CI (60.7 to 62.7); mean APACHE II 12.6 (SD ±8.42), CI (12.12 to 13.11); and a mean time stay of 4.84 days (SD ±6.26). In total, 68.9% were provided from the community. A total of 44.1% were coronary, 2.84% trauma and 53.02% medical-surgical patients. A total of 29.8% had antibiotic therapy in the ICU, 20% had it before incoming. In total, 18.38% were treated with artificial airway (MV, tracheostomy). In total, 54.09% used a urinary catheter and 38.8% needed a central venous catheter. Fifteen percent of patients had some kind of surgery before admission; 4.8% required the extrarenal depuration technique. In total, 497 patients (44.1%) were coronary, 49.5% male, mean age 66.18 (SD ±12.6), CI (64.88 to 67.48); mean APACHE II 9.74 (SD ± 6.1), CI (9.1 to 10.3); and a mean time stay of 3.62 days (SD ±4.7), CI (3.1 to 4.1). Mortality in this group was 3.7%. In 61.9% of cases the diagnosis of admission was AMI, 16% arrhythmia and 11.6% unstable angina. Of patients, 629 were polyvalent (55.8%), 53.85% male, mean age 58.05 (SD ±17.2), CI (56.7 to 59.4); mean APACHE II 14.6 (SD ±9.1), CI (13.8 to 15.3); and a mean time stay of 4.64 days (SD ±7.7), CI (4 to 5.25). Mortality was 11.6%. In 33.2% the cause of income was digestive, 23.2% acute or chronic exacerbated respiratory failure, 12.4% severe sepsis/septic shock and 10.1% postoperative cardiovascular surgery. The incidence density (ID) of catheter-related bacteremia was 5.5, 92.8% from the fourth day of ICU admission; ID of ventilator-associated pneumonia (VAP) was 5.94, 88.9% since the fourth day; and ID of urinary tract infections (UTI) related to urinary catheter was 2.88, 80% of them since the fourth day. From all patients who developed intra-ICU infections, mean APACHE II in admission was 21.3 (SD ±9.6) with a mean time stay of 23.4 days (SD ±12.9) and a mortality percentage of 19.6%.

## Conclusion

In our ICU the main cause of admission was the polyvalent patient, who is younger and has more severity with not much difference in mean time of stay compared with the coronary patient. The intra-ICU infections provide an increase of morbi-mortality risk and consumption of resources.

